# Constitutive activity of NF-kappa B in myeloid cells drives pathogenicity of monocytes and macrophages during autoimmune neuroinflammation

**DOI:** 10.1186/1742-2094-9-15

**Published:** 2012-01-20

**Authors:** Gisa Ellrichmann, Jan Thöne, De-Hyung Lee, Rudolph A Rupec, Ralf Gold, Ralf A Linker

**Affiliations:** 1 Department of Neurology, St. Josef Hospital Bochum, Ruhr-University Bochum, Germany; 2 Department of Dermatology, Ludwig-Maximilian-University Munich, Germany; 3 Department of Neurology, Friedrich-Alexander-University Erlangen, Germany

**Keywords:** NF-kappaB, myeloid cells, cytokines, experimental autoimmune encephalomyelitis

## Abstract

The NF-κB/REL-family of transcription factors plays a central role in coordinating the expression of a wide variety of genes controlling immune responses including autoimmunity of the central nervous system (CNS). The inactive form of NF-κB consists of a heterodimer which is complexed with its inhibitor, IκB. Conditional knockout-mice for IκBα in myeloid cells (lysMCreIκBα^fl/fl^) have been generated and are characterized by a constitutive activation of NF-κB proteins allowing the study of this transcription factor in myelin-oligodendrocyte-glycoprotein induced experimental autoimmune encephalomyelitis (MOG-EAE), a well established experimental model for autoimmune demyelination of the CNS.

In comparison to controls, lysMCreIκBα^fl/fl ^mice developed a more severe clinical course of EAE. Upon histological analysis on day 15 p.i., there was an over two fold increased infiltration of T-cells and macrophages/microglia. In addition, lysMCreIκBα^fl/fl ^mice displayed an increased expression of the NF-κB dependent factor inducible nitric oxide synthase in inflamed lesions. These changes in the CNS are associated with increased numbers of CD11b positive splenocytes and a higher expression of Ly6c on monocytes in the periphery. Well in accordance with these changes in the myeloid cell compartment, there was an increased production of the monocyte cytokines interleukin(IL)-12 p70, IL-6 and IL-1beta in splenocytes. In contrast, production of the T-cell associated cytokines interferon gamma (IFN-gamma) and IL-17 was not influenced.

In summary, myeloid cell derived NF-κB plays a crucial role in autoimmune inflammation of the CNS and drives a pathogenic role of monocytes and macrophages independently from T-cells.

## Background

Multiple sclerosis (MS) is the most common human demyelinating disease of the central nervous system (CNS). The development of autoimmune diseases such as MS requires the coordinated expression of a number of pro-inflammatory genes. These factors may influence the activation, migration and effector function of inflammatory cells and encompass a variety of cytokines, chemokines, adhesion molecules as well as other inflammatory factors.

Nuclear factor (NF-) kappaB (NF-κB) is essential for both innate and adaptive immunity [1]. NF-κB is an inducible transcription factor which is detected in most cell types and is involved in many inflammatory processes. It consists of homo- or heterodimers of different subunits and structurally related proteins (Rel/NF-κB-proteins). There are at least five Rel/NF-κB proteins: c-Rel, RelA (p65), RelB, NF-κB1 (p50/p105), NF-κB 2 (p52/p100) [1-4]. The transcriptional activation of the NF-κB pathway is controlled by the inhibitor of NF-κB, IκB. IκB is phosphorylated by IκB kinase (IKK), a complex that is composed of a regulatory subunit IKK-γ. Polyubiquitinylation of IκB induces NF-κB dimers to translocate to the nucleus, inducing the transcription of over 150 target genes [5].

Besides the involvement of NF-κB in T-cell proliferation and activation [6-8], it is also a key element in coordinately controlling gene expression during monocyte/macrophage activation [9]. In particular the macrophage-derived cytokines interleukin-1beta (IL-1 β) and tumor necrosis factor-alpha (TNF-α), are potent activators of NF-κB. In turn, their expression is controlled by NF-κB thus resulting in a positive feedback loop. Hence, NF-κB signalling pathways may play a pivotal role in activating myeloid cell function during autoimmune inflammation. In addition to its central mediatory function in cytokine expression, NF-κB in myeloid cells may be induced by physical as well as oxidative stress to cells, e.g. via the inducible nitric oxide synthase (iNOS) [10] or cyclooxygenase-2 (COX-2) [11].

In our study, we investigated the role of NF-κB in myeloid cells during autoimmune demyelination of the CNS. For the targeted analysis of NF-κB functions in monocytes/macrophages, conditional knockout-mice for IκBα in myeloid cells (lysMCreIκBα^fl/fl ^mice) have been generated [12]. These mice display a constitutive expression of NF-κB proteins in macrophages and monocytes, but no spontaneous myelopoetic phenotype thus allowing for studying the role of this transcription factor in myelin-oligodendrocyte-glycoprotein induced experimental autoimmune encephalomyelitis (MOG-EAE). Our results demonstrate that NF-κB-dependent pro-inflammatory gene expression in monocytes and macrophages plays an important role for CNS pathology in autoimmune neuroinflammation. In turn, targeting the IKK-NF-κB pathway in myeloid cells might constitute an interesting therapeutic target in MS.

## Methods

### Animals

Conditional knockout-mice for IκBα in myeloid cells (lysMCreIκBα^fl/fl ^mice) have been generated at the Ludwigs-Maximilians-University, Munich, Germany [12] and were backcrossed to the C57BL/6 background for more than 10 generations. Complete inactivation of IκBα results in hypergranulopoiesis and perinatal death. Therefore the Cre-loxP recombination system was used to generate a mouse line that allows for selective deletion of IκBα. The targeting construct was designed in a way that Cre-mediated recombination results in deletion of the promoter region containing essential regulatory NF-κB binding sites. These conditional IκB knockout mice displayed constitutively high nuclear levels of NF-κB in myeloid cells. C57BL/6 mice for backcrossing and controls were bred in the same mouse colony. All mice were housed under pathogen free conditions at the animal facility of the Ruhr-University Bochum, Germany. All experiments have been reviewed and approved by the North-Rhine-Westphalia authorities for animal experimentation. Mice were given food and water at libidum and were weighed daily to obtain weight curves. All animal experiments were approved by the local authorities for animal experimentation (approval ID: 50.8735.1 Nr. 114/6, § 8 Protection of Animals Act). For survival analysis, cohorts of mice were followed over the course of disease with moribund mice sacrificed according to animal protection laws.

### Induction of EAE

Ten-week-old C57BL/6 female mice and lysMCreIκBα^fl/fl ^knockout mice were immunized via subcutaneous injection of 200 μg MOG_35-55 _peptide (Charité, Berlin, Germany) in complete Freund's adjuvant containing 200 μg Mycobacteria tuberculosis (Difco/BD Biosciences, Heidelberg, Germany). All mice received 200 ng pertussis toxin (List/Quadratec, UK) on days 0 and 2 post immunization by intraperitoneal injection.

Mice were weighed and scored for clinical signs daily according to a 10-scale score as described previously [13].

### Splenocyte culture and proliferation assay

For cytokine assays, splenocytes were cultured at 6 × 10^6 ^cells/well in 24 well plates containing 1 ml in RPMI medium/well (Gibco, Eggenstein, Germany) with various concentrations of MOG_35-55 _peptide, or ConA (1.25 μg/ml). For the criss-cross proliferation assay, a MACS^® ^cell separation (Pan-T-cell kit, Milentyi) for T-cells was used. Both pooled APC and T-cells were isolated form MOG-immunized mice on day 10 after immunization. Culture supernatants for cytokine analysis were collected 48 h later. For proliferation assays, 0.3 × 10^6 ^cells/well were used in 96 well plates. [^3^H]thymidine was added to each culture at 48 h and cells were harvested 16 h later. Data are depicted as stimulatory index indicating the fold increase in [^3^H]thymidine incorporation as compared to medium only.

### Histological analyses

Mice were deeply anesthetized with ketamine and transcardially perfused with 4% paraformaldehyde in phosphate buffered saline (PBS, pH 7.4) for light microscopy and immunohistochemistry.

Spinal cord was removed, embedded in paraffin and routinely processed to obtain 12 cross sections per mouse. Routine stainings comprised cresyl violet staining, Luxol Fast Blue for myelin and Bielschowsky silver impregnation for axons. Immunohistochemistry including iNOS staining (1:200; Millipore, Schwalbach, Germany) was performed on 3 μm thick paraffin sections. All other histological procedures were essentially performed as described previously [13]. Briefly, T-cells were labeled by rat anti-CD3 (Serotec; Wiesbaden, Germany; 1: 300), macrophages/microglia by rat anti-mouse Mac-3 (BD Heidelberg, Germany; 1:200) and neutrophils by rat anti 7/4 antigen (Serotec; 1:300).

### Assay of cytokines by ELISA

Cell culture supernatants were collected, aliquoted and stored at -20°C. Immediately before measurement, aliquots were brought to room temperature and analyzed for Interleukin (IL) IL-1β, IL-6, IL-12(p40), IL-12(p70), IL-17 and interferon gamma (IFN-γ) using sandwich ELISA kits (BD and R&D Systems, Minneapolis, USA) according to manufacturers' protocols.

### CD11b cell isolation and FACS analysis

Splenic CD11b+ cells were isolated by magnetic cell sorting (MACS) according to manufacturer's protocols (Miltenyi Biotec, Auburn, USA). The purification level of CD11b+ exceed routinely 95%. The expression of CD11b, CD80, CD86, MHC class II (MHC-II) and Ly6c was evaluated both prior and subsequent to MACS using a FACS Canto II and CellQuest software (BD, Heidelberg, Germany). Monoclonal antibodies were all purchased from BD.

### Quantification and statistical analyses

All neuropathological and scoring analyses were performed completely blinded. All data are presented as mean ± SEM. Histological quantification was performed by means of overlaying a stereological grid onto the sections and counting T-cells, macrophages/microglia, neutrophils, iNOS positive profiles and axonal densities on 6 lesions per mouse from representative spinal cord cross sections comprising cervical, thoracic and lumbar spinal cord as described previously [13]. Axonal densities were counted using a 24 point eyepiece from Olympus (Hamburg, Germany), and the number of points crossing axons was measured as a fraction of the total number of points on the sterological grid [14]. Demyelination was analyzed semi-automatically with the CellD software (Olympus).

Statistical analysis was performed by Mann-Whitney U-test for histological evaluations and clinical course, by t-test, for ELISA and FACS analyses and by two-way ANOVA with Dunn's post test for proliferation assays (all analyses done by Graph Pad Prism 5, San Diego, CA, USA). In all experiments, a probability level of *p < 0.05, **p < 0.01, ***p < 0.001 was considered to be statistically significant.

## Results

### Characterization of mice with a conditonal IκB deletion in myeloid cells

**Figure 1:**
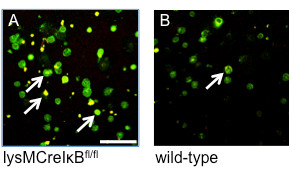
**Constitutive activation of NF-κB in lysMCreIκBα^fl/fl ^monocytes**. Representative cytospins of naïve CD11b-sorted spleen monocytes from lysMCreIκBα^fl/fl ^mice (A) or wild-type controls (B) after staining for the NF-κB p65 subunit and nuclear co-staining with TOTO-3. Note the accumulation of active NF-κB in the nucleus of monocytes from a representative lysMCreIκBα^fl/fl ^mouse (marked by arrows). Bar = 20 μm.

In this study, we employ lysMCreIκBα^fl/fl ^mice to analyze the role of NF-κB in monocytes during autoimmune inflammation. While these mice have been characterized in detail before [12], we analyzed the expression of the active, phosphorylated p65 NF-κB subunit in naive monocytes by confocal laser scanning microscopy (Figure 1). To this end, CD11b-positive monocytes from lysMCreIκBα^fl/fl ^or wild-type control mice were isolated and used for anti-NF-κB-p65 immunocytochemistry. Double label experiments with TOTO-3 as nuclear staining revealed that naive monocytes from lysMCreIκBα^fl/fl ^mice showed constitutive activity of p65 in monocytes while there was no NF-κB activation in naive monocytes from wild-type controls. In summary, naive monocytes from lysMCreIκBα^fl/fl ^mice are characterized by an increased activity of the NF-κB system and constitute a useful tool to study the role of NF-κB in monocytes during autoimmune inflammation.

**Figure 2:**
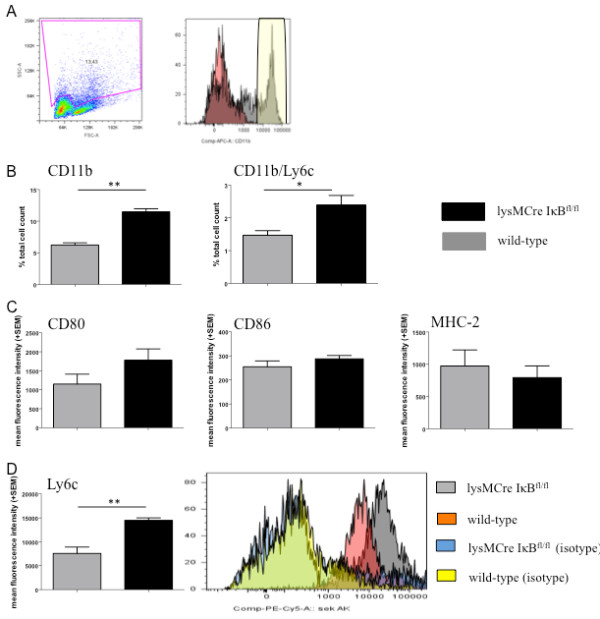
**FACS phenotying of lysMCreIκBα^fl/fl ^mice**. (A) Analysis of spleen cells from naïve lysMCreIκBα^fl/fl ^mice (n = 7) and wild-type mice (n = 8). Isotype controls are coloured in red, CD11b+ cell fractions are marked with a yellow box. (B) FACS analyses show a significantly increased number of spleen-derived CD11b positive monocytes and CD11b/Ly6c double positive cells in lysMCreIκBα^fl/fl ^mice. Data are shown in per cent of total cell count. (C) lysMCreIκBα^fl/fl ^mice display a non significant increase of the co-stimulatory molecules CD80 and CD86. While there is no effect on MHC-II (C), there is a significant increase in Ly6c expression on CD11b+ cells from lysMCreIκBα^fl/fl ^mice (D).

We next investigated the functional impact of the conditonal IκB deletion in naive CD11b positive splenocytes and in particular CD11b/Ly6c positive monocytes via FACS (Figure 2A, B). We observed a significantly higher number of spleen-derived CD11b positive cells and a higher total cell count of CD11b/Ly6c double positive cells in spleens from lysMCreIκBα^fl/fl ^mice (Figure 2B, Table 1). FACS analyses of CD11 positive cells after MACS sorting did not reveal any significant differences in the expression of CD80, CD86 and MHC-II (Figure 2C). However, expression of Ly6c was significantly increased on CD11b cells from lysMCreIκBα^fl/fl ^mice (Figure 2D).

**Table 1:** FACS analyses.

	lysMCreIκBα^fl/fl^	wild-type	*p*-value	n
**% of CD11b positive spleen cells **[± SEM]	11.5 ± 1.3	6.2 ± 1.0	0.0003	7/8
**absolute numbers of CD11b positive spleen cells **[± SEM]	2299.7 ± 98	1246 ± 69	0.0003	7/8

Quantification of CD11b positive spleen cells. 20.000 cells/mouse were analyzed.

### Constitutive activation of NF-κB in myeloid cells leads to a more severe course of MOG-EAE with enhanced inflammatory infiltration and demyelination

**Figure 3:**
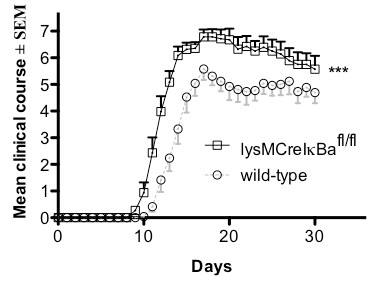
**The clinical course of MOG-EAE in lysMCreIκBα^fl/fl ^mice is more severe**. Clinical course of EAE in wild-type mice (n = 13) and lysMCreIκBα^fl/fl ^mice (n = 14). lysMCreIκBα^fl/fl ^mice (black curve) show a significantly earlier start and a more severe course of disease compared to wild-type mice (grey curve). Data represent the mean of two independent experiments ± SEM.

To investigate the role of a constitutive activation of NF-κB in myleoid cells during autoimmune neuroinflammation, we analyzed clinical symptoms and disease severity of MOG-EAE in lysMCreIκBα^fl/fl ^mice as compared to age and gender matched wild-type control mice (Figure 3). Disease incidence and mortality from EAE did not differ between groups (wild-type mice n = 13 and lysMCreIκBα^fl/fl ^mice n = 14). However, lysMCreIκBα^fl/fl ^mice displayed a significantly more severe clinical course of EAE. On day 20 post immunization, conditional knock-out mice suffered from severe paraparesis while mice in the control group only displayed severe gait ataxia.

**Figure 4:**
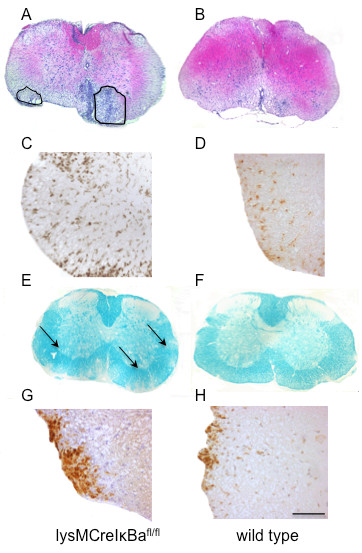
**Enhanced inflammatory infiltration, demyelination and iNOS expression in lysMCreIκBα^fl/fl ^mice**. Histological analyses of lysMCreIκBα^fl/fl ^mice (A, C, E, G) versus wild-type mice (B, D, F, H) result in increased inflammatory infiltration after HE staining or Mac-3 staining (A, B, C, D) and increased demyelination after Luxol Fast Blue staining (E, F). As compared to wild-type controls, lysMCreIκBα^fl/fl ^mice are also characterized by an increase in iNOS expression cells (G, H). Representative images of spinal cord cross section in the acute phase on day 15 post immunization are shown. Infiltrated areas are marked in the knockout-mice group (A). Scale bar is only depicted in (H) for clarity and represents 200 μm for A, B, E, F and 20 μm in C, D, G, H.

Next, we focused on histological analyses to determine whether the clinical differences between groups correlated with enhanced infiltration of inflammatory cell subsets in the CNS as well as demyelination and axonal damage. Upon histological analysis of spinal cord cross sections on day 15 post immunization, there was an increased number of inflammatory infiltrates and an increased infiltration of CD3 positive T-cells as well as Mac-3 macrophages/microglia and an increased amount of iNOS positive cells in lysMCreIκBα^fl/fl ^mice (Table 2 and Figure 4). The analysis of myelin loss after Luxol Fast Blue staining showed a significant increase in demyelination in lysMCreIκBα^fl/fl ^mice (Figure 4). Upon evaluation of axonal densities after Bielschowsky silver impregnation as well as numbers of iNOS positive cells and infiltrating 7/4-antigen positive neutrophils, there was a trend towards enhanced axonal injury as well as an increased neutrophil infiltration in lysMCreIκBα^fl/fl ^mice, but without statistical significance (Table 2).

**Table 2:** Histological analyses.

	lysMCreIκBα^fl/fl^	wild-type	*p*-value	n
**Inflammatory Index **[± SEM]	16.0 ± 1.9	6.8 ± 1.0	0.0003	5/5
**CD 3 **[cells/mm^2 ^± SEM]	251 ± 19	102 ± 13	< 0.0001	5/5
**Mac-3 **[± SEM]	1123 ± 81	581 ± 100	< 0.0001	5/5
**iNOS **[± SEM]	333 ± 75	153 ± 19	0.0184	5/5
**% Demyelination **[± SEM]	8 ± 0.8	2 ± 0.6	< 0.0001	5/5
**Neutrophils **[± SEM]	339 ± 37	190 ± 25	n.s	5/5
**Axonal densities **[± SEM]	3.7 ± 0.5	2.9 ± 0.3	n.s	5/5

Blinded quantification of the inflammatory index after HE-staining, CD3 positive cells, Mac-3 positive macrophages/microglia, iNOS positive cells, demyelination after Luxol Fast Blue staining and axonal densities after silver impregnation were performed on day 15 p.i. marking the acute phase of the disease. Data are presented as cells/mm^2 ^for CD3, Mac-3, iNOS positive cells, percent demyelination for Luxol Fast Blue and relative axonal densities for silver impregnated axons. n.s = not significant.

We also examined inflammatory infiltration at a later time point (day 30 p.i.) which revealed less inflammation, but a similar trend towards enhanced inflammatory infiltration in lysMCreIκBα^fl/fl ^mice (data not shown).

### Constitutive activation of NF-κB in myeloid cells results in increased monocyte cytokine production

**Figure 5:**
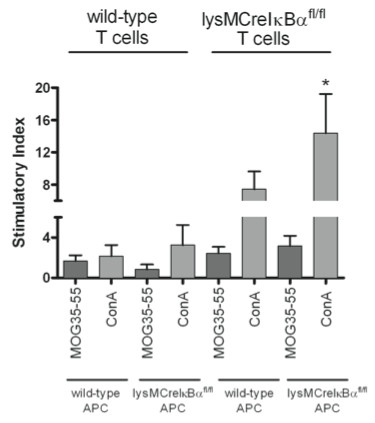
**Increased T-cell proliferation with APC from lysMCreIκBα^fl/fl ^mice**. Analysis of T-cell proliferation in splenocyte primary cultures from MOG-immunized mice after recall with MOG antigen. Data from a representative splenocyte culture experiment with n = 4 for each group are shown. All data are depicted as stimulatory index and expressed as mean ± SEM. Note the increased T-cell proliferation specifically after culture of lysMCreIκBα^fl/fl ^T-cells with lysMCreIκBα^fl/fl ^mice APC and after stimulation with ConA.

**Figure 6:**
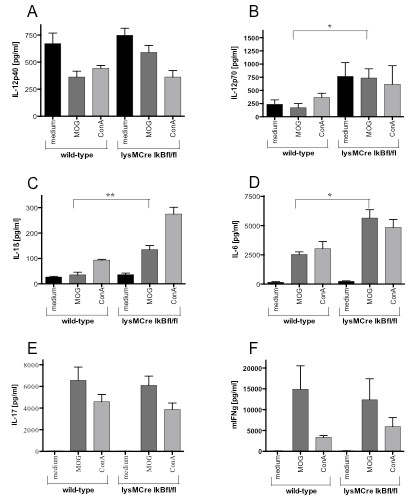
**Increased expression of NF-κB dependent monocyte/macrophage cytokines in splenocyte primary culture**. ELISA based analysis of cytokines in a MOG_35-55 _recall assay from splenocyte cultures. Data from a representative splenocyte culture experiment with n = 4 for each group are shown. All data are expressed as mean ± SEM. While production of interleukin IL-12p40 (A) is similar between both groups, lysMCreIκBα^fl/fl ^mice are characterized by an increased production of the NF-κB dependent cytokines IL-12p70 (B), IL-1β (C) and IL-6 (D). In contrast, production of the T-cell cytokines IL-17 (E) and interferon gamma (IFN-γ, F) is not influenced.

Since we found a more severe EAE with enhanced inflammatory infiltration in lysMCreIκBα^fl/fl ^mice, we next examined the involved immunological processes in this model. To this end, we analyzed T-cell proliferation and cytokine production in splenocyte primary cultures from MOG-immunized mice after recall with MOG antigen. In co-culture experiments of wild-type T-cells with lysMCreIκBα^fl/fl ^antigen presenting cells and vice versa, we found an increased T-cell proliferation specifically after culture of lysMCreIκBα^fl/fl ^T-cells with lysMCreIκBα^fl/fl ^APC and after stimulation with ConA (Figure 5). Upon analysis of IL-1β, IL-6, and IL-12(p40/70) after re-stimulation with MOG in splenocyte culture (Figure 6A-D), there was a significant increase of the NF-κB dependent monocyte cytokines IL-1β, IL-6 and IL12p70 in the supernatant from lysMCreIκBα^fl/fl ^mice as compared to controls (Figure 6B-D). In contrast, production of the T-cell cytokines IL-17 and IFN-γ was not influenced (Figures 6E, F).

In summary, lysMCreIκBα^fl/fl ^mice displayed an altered monocyte cytokine profile which was associated with an increased production of IL-1β, IL-6, and IL-12(p70) in splenocyte culture.

## Discussion

Here we analyze the role of monocyte/macrophage derived NF-κB in autoimmune demyelination. We studied EAE in mice which are devoid of IκBα in myeloid cells (lysMCreIκBα^fl/fl ^mice). In these mice, loss of IκB in monocytes and macrophages leads to constitutive expression of NF-κB. In turn, this results in an increased expression of NF-κB regulated monocyte/macrophage cytokines and subsequently enhanced macrophage infiltration and iNOS expression in the spinal cord of EAE mice. These mechanims govern demyelination, enhanced axonal damage and finally a more severe course of MOG-EAE. Thus macrophage derived, NF-κB dependent cytokines may play a pivotal role in the pathogenesis of EAE and determine the outcome of autoimmune inflammation in the CNS without interfering with Th1 and Th17 T-cell responses. Our findings suggest that NF-κB in myeloid cells is a master regulator for regulation of inflammation and tissue damage in autoimmune inflammation of the CNS.

Previous studies already investigated the role of NF-κB in the CNS [15], in autoimmune diseases [16] and also during EAE. The analysis of c-Rel or NF-κB1-deficient mice as well as IKK-2-deficient mice revealed that NF-κB activation in T-cells significantly contributes to the initiation of autoimmune neuroinflammation [17,18]. Myelin-specific T-cells in NF-κB1 (p50)-deficient mice are deficient in differentiating into either Th1 or Th2 cells [17]. This concept was further refined by Dasgupta and co-workers who showed positive effects of NF-κB on the differentiation of myelin-specific Th1 cells, but a negative influence on the differentiation into Th2 cells [19]. In line with these observations, severely impaired T-cell responses were found in immune cell cultures derived from mice with a T-cell specific deficiency in IKK2, a pivotal kinase for NF-κB activation (IKK2^Δ T-cell ^mice) [20].

Moreover, NF-κB may also play a role in the CNS cells during autoimmune demyelination. While studies by Wooten or Mattson and co-wokers in cell culture argue for a protective role of NF-κB in neuronal cells [21,22], our work with conditional overexpression of NF-κB in myeloid cells reveal monocytes/macrophages as a further cell type with crucial importance of NF-κB in neuroinflammation.

Many studies have demonstrated a critical role for macrophages/microglia as well as an up-regulation of MHC class II in these cells in EAE and MS lesions [23,24]. Depletion experiments with liposomal dichloromethylene diphosphonate (Cl_2_MDP) revealed a crucial role of bone marrow derived macrophages for tissue damage in EAE [25,26]. Interestingly, histological analyses in these studies could not detect differences in number and localization of CNS infiltrating T-cells between Cl_2_MDP treated rats and controls [25]. These data argue for a myeloid cell independent T-cell activation in EAE. Well in line with this concept, we did not observe any change in T-cell cytokine expression in our model. Indeed, not monocytes/macrophages, but rather dendritic cells as professional antigen presenting cells do play the major role for T-cell activation in EAE [27]. Furthermore, lysMCre mediated IκBα deletion in our model does not involve dendritic cells and does not lead to effects on T-cell cytokines. Thus at first glance, NF-κB in macrophages/monocytes may be more important for effector functions than for T-cell activation. Yet, changes in phagocyte cytokine patterns may eventually result in an enhanced T-cell activation over time which is not reflected in our analysis at day 10 after immunization. Further studies are warranted to dissect indirect effects of myeloid cell derived NF-κB on T-cell function in the setting of chronic inflammation.

Our data reveal a crucial role of myeloid cell derived NF-κB for demyelination. In our model, demyelination or oligodendrocyte apoptosis may be mediated by a direct phagocytic attack of macrophages or also by macrophage derived toxic cytokines. Here, especially TNF-α as a typical NF-κB regulated cytokine in macrophages may play a role [28]. Previous studies already indicated a major role of TNF-α for macrophage recruitment from the periphery [29] and also for oligodendrocyte apoptosis as well as toxic demyelination, especially in the MOG-EAE models of the C57BL/6 mouse [30,31]. Finally, myelin loss might also be influenced by free radicals. As shown, overexpression of NF-κB may lead to an increased expresion of the NF-κB target gene inducible NO-synthase (iNOS) and thus an increased NO production which was previously shown to exert a detrimental role on oligodendrocytes and in EAE [32,33].

Moreover, reactive oxygen species (ROS) intermediates may induce cellular damage and trigger demyelination as well as a recently shown reversible axonal damage called focal axonal degeneration (FAD) [34]. In this process, ROS mediated NF-κB activation [35] and in turn NF-κB induced further ROS production in macrophages may play an important role. This notion is underscored by the fact that macrophages were found to play an important role in the process of ROS mediated FAD [34].

In our model, further pathways in NF-κB activation may be involved. Among others, these factors may include toll-like receptors (TLR). In EAE, especially TLR9 on microglia may play an important role for interaction with invading immune cells and for the initiation of a cascade resulting in NF-κB activation and finally an enhanced innate immune responses [36].

Theoretically, NF-κB overexpression in macrophages may also exert protective effects in EAE. Indeed, overexpression of the NF-κB target gene "triggering receptor expressed on myeloid cells 2" (TREM2) in myeloid cells lead to an enhanced expression of anti-inflammatory cytokines in the spinal cord of EAE mice as well as a reduced amount of demyelination and axonal damage [37]. In our setting, overexpression of NF-κB in macrophages predominantly induces destructive effects thus arguing for the prevailing importance of NF-κB mediated pathways for detrimental phagocyte functions in EAE.

## Conclusions

In summary, our results identify immune-mediated pathways in myeloid cells and in particular monocyte/macrophage derived NF-κB as a potential target for therapeutic interventions in the treatment of autoimmune diseases such as MS. This concept is well in line with previous studies using lysMCre mediated conditional gene targeting in macrophages. The myeloid cell specific deletion of the type I interferon receptor also revealed these phagocytes as an interesting therapeutic target in autoimmune neuroinflammation [38].

## Competing interests

The authors declare that they have no competing interests.

## Authors' contributions

GE carried out the experiments, analyzed histological and immunological results and drafted the manuscript. JT supported cytokine assays and interpretation of results. DHL carried out the confocal laser scanning microscopy. RAR generated the knock-out mice. RG provided general support and participated in the design of the study. RAL conceived the study, and participated in its design and coordination and surveyed and supported the draft of the manuscript. All authors read and approved the final manuscript.
